# G protein–coupled receptor kinase 3 regulates CCR7 signaling, trafficking, and T cell responses to CCL19 and CCL21

**DOI:** 10.1016/j.jbc.2026.113466

**Published:** 2026-08-19

**Authors:** Anahi Sanchez, Cesar I. Cardona, Brian Kaiser, Charles A. Bill, Mary E. Miller, Daniel W. Bassuk, Brant M. Wagener, Michael A. Brissette, Dalilia Ibanez, Manuel Llano, Colin A. Bill, Charlotte M. Vines

**Affiliations:** 1 Department of Biological Sciences, The University of Texas at El Paso, El Paso, Texas, USA; 2 Department of Microbiology, Molecular Genetics and Immunology, The University of Kansas Medical Center, Kansas City, Kansas, USA; 3 Department of Internal Medicine, Texas Health Hospital, Texas Health Physicians Group, Fort Worth, Texas, USA; 4 Department of Anesthesiology and Perioperative Medicine, University of Alabama Birmingham School of Medicine, Birmingham, Alabama, USA

**Keywords:** Chemokine, G protein-coupled receptor, arrestin, migration, trafficking

## Abstract

G protein–coupled receptors (GPCRs) can bind multiple ligands leading to differential signaling events termed biased signaling. GPCR kinases (GRKs) can control the phosphorylation patterns of a GPCR to regulate biased signaling. Since the functions of GRKs are dependent on the local cellular environment, our study focused on investigating the roles of GRK3 in regulating biased and overlapping signaling events downstream of CCR7 activation by its ligands CCL19 and CCL21 in T cells, where CCR7 is expressed endogenously. We found stable CCR7 internalization, Gα_i3_ activation, and β−arrestin recruitment were regulated by GRK3 downstream of CCL19 activation of CCR7. In contrast, while CCL21 does not promote recruitment of β-arrestins, GRK3 had no effect on internalization of CCL21 bound CCR7, or its ability to activate Gα_i1,_ Gα_i2_ or Gα_i3_. We also found that GRK3 limited T cell migration and Ca^2+^ mobilization in response to both CCL19 and CCL21. Since misregulation of CCR7 and its ligands have been linked to autoimmune disease, these studies could lead to a better understanding of mechanisms that promote homeostasis or pathogenesis.

G protein–coupled receptors (GPCRs), the largest class of receptors in humans, initiate signaling events that are essential for maintaining individual health and overall physiological balance. Given their roles in promoting homeostasis, these receptors are the targets of ∼30% of drugs approved by the United States Food and Drug Administration (1, 2). Following ligand binding GPCRs are activated to regulate diverse cellular responses and can be desensitized through recruitment of GPCR kinases (GRKs) that phosphorylate GPCRs, and recruitment of β-arrestins, which block activation of G proteins (3). Different signaling events, termed biased signaling, can take place downstream of GPCR phosphorylation or β-arrestin binding (4).

GRKs can control the biased signaling following ligand-binding, by catalyzing specific phosphorylation patterns or barcodes of a GPCR that in turn, control unique downstream signaling events. Barcodes regulate distinct effectors that can interact with each GPCR on its cytoplasmic face (5, 6). The phosphorylation pattern, which can vary dependent upon the GRK that is activated, can regulate recruitment of additional signaling molecules such as E3 ubiquitin ligases to control protein stability and β-arrestins which can determine endosome localization and recruitment of mitogen-activated protein-kinases (MAPKs) (7, 8, 9, 10, 11, 12, 13, 14). Yet, while GRKs play pivotal roles in GPCR signaling, their roles in biased signaling are not well understood.

Mammalian cells express GRKs that are classified based on sequence homology (15). Along with other kinases, GRKs can phosphorylate serine or threonine amino acids, primarily on the third intracellular loop and C terminus of GPCRs to promote heterologous desensitization of a GPCR (16). GRKs are subdivided into three families. The GRK1 subfamily, which consists of GRK1 and 7 are visual GRKs found in the human retinal cones and rods (17, 18). The GRK2 subfamily consists of GRK2 and GRK3, and the GRK4 subfamily includes GRK4, GRK5, and GRK6. GRK2, 3, 5 and 6 are ubiquitously expressed in all human tissues, yet of these abundantly expressed GRKs, only GRK2 and GRK3, which are localized to the cytoplasm, are activated exclusively by binding active GPCRs (19). Ligand mediated activation of the GRK2 subfamily by a GPCR, is dependent on engagement *via* the GRK pleckstrin homology domain with Gβγ, anchoring the GRK to the membrane and expediting phosphorylation of the GPCR, subsequently recruiting a β-arrestin and initiating receptor internalization (19, 20). This association implicates GRK2 family members in regulating the stoichiometry of available heterotrimeric G proteins. Although multiple GRKs can phosphorylate GPCRs, differences of GRK expression in different tissues can affect the response of GPCRs as well (21). Therefore, defining the function of each GRK, cell type specific information can be ascertained by examining endogenous GPCR/GRK relationships, in the cells, in which they are expressed.

C-C chemokine receptor 7 (CCR7), belongs to a family of chemokine binding GPCRs that promotes migration of thymocytes during development, and of naïve T cells and other immune cells (22, 23) during homeostasis and inflammation (24). In T cells, following activation by its ligand C-C chemokine 19 (CCL19), CCR7 is phosphorylated at high levels, and rapidly internalized (25). In contrast, in response to binding to its other ligand, CCL21, there is minimal phosphorylation of the C terminus in T cells, and low levels of receptor internalization (26). We have shown that this promotes differential T cell signaling events (26, 27, 28, 29, 30) and at times overlapping signaling events. This type of differential signaling is termed biased signaling (31). Although GRKs play important roles in GPCR functions, the contributions of GRKs to biased signaling of CCR7 in T cells is not well understood.

CCR7 can be phosphorylated on the C terminus by GRKs (3). Although endogenous GRK3 and GRK6 have been shown to phosphorylate exogenous CCR7 in response to CCL19 in human embryonic kidney (HEK) cells overexpressing CCR7, only GRK6 promoted CCR7 phosphorylation in the presence of CCL21 (3). In contrast, in a second study which focused on the roles of GRK2 and GRK6 in primary murine lymphocytes, revealed that chemotactic migration of cells to CCL19 or CCL21, was unaffected in T lymphocytes in the absence of GRK2 or GRK6, while in B lymphocytes, deletion of GRK2 reduced migration to CCL19, although deletion of GRK6 enhanced migration to the same ligand. Deletion of GRK2 or GRK6 had no effect on B cell migration to CCL21 (32). Since the contribution of GRK to receptor deactivation is determined by the local concentrations of GRKs within a cell (21), our goal was to determine if endogenous GRK3 regulated CCR7 function in T cells, to regulate differential receptor internalization and downstream signaling.

GPCR phosphorylation can regulate β-arrestin recruitment, and the resultant conformational changes in the structure of β-arrestin, which promote downstream signaling (33, 34). There are four members of the arrestin family; arrestin 1 and 4 expression that are limited to expression in the retina, and arrestin 2 and 3 (β arrestin 1 and β arrestin 2, respectively) which are ubiquitously expressed. CCL19 promotes CCR7 phosphorylation in peripheral T cells and activation of ERK1/2 and ERK5 (25, 26, 27); this internalization of CCR7/CCL19 is dependent upon arrestin 3 (26). Yet the question of whether GRKs or a different group of kinases control differential biased signaling of CCR7 in T cells has remained unclear.

In this study, we find that homozygous deletion of GRK3 in mice, results in a significant decrease in receptor phosphorylation independent of ligand. In contrast, disruption of GRK3 in human T cells leads to a significant decrease in receptor phosphorylation, only in the presence of CCL21. Further, although both CCR7/CCL19 and CCR7/CCL21 internalize in the absence of GRK3, recruitment of β-arrestin to the ligand bound, internalized receptor is delayed in the CCR7/CCL19 complex in the absence of GRK3, demonstrating a role for this GRK3 in biased recruitment of β-arrestins. Chemokine receptors signal *via* Gα_i_, of which there are three different subtypes (Gα_i1,_ Gα_i2_ and Gα_i3_ (35, 36)). Using a fluorescence resonance energy transfer (FRET) assay, we found that GRK3 limits activation of Ga_i3_. Examination of cell physiological responses reveals a role for GRK3 in limiting signaling *via* Ca^2+^ mobilization and downstream T cell migration. This is the first description of a role for GRK3 in regulating biased signaling in T cells to control receptor activation of G proteins, receptor internalization and recruitment of β−arrestins, while limiting chemotactic migration and Ca^2+^ mobilization in T cells.

## Results

### GRK3 differentially regulates CCR7 phosphorylation and affects CCR7 internalization in murine splenic T cells

**Figure 1:**
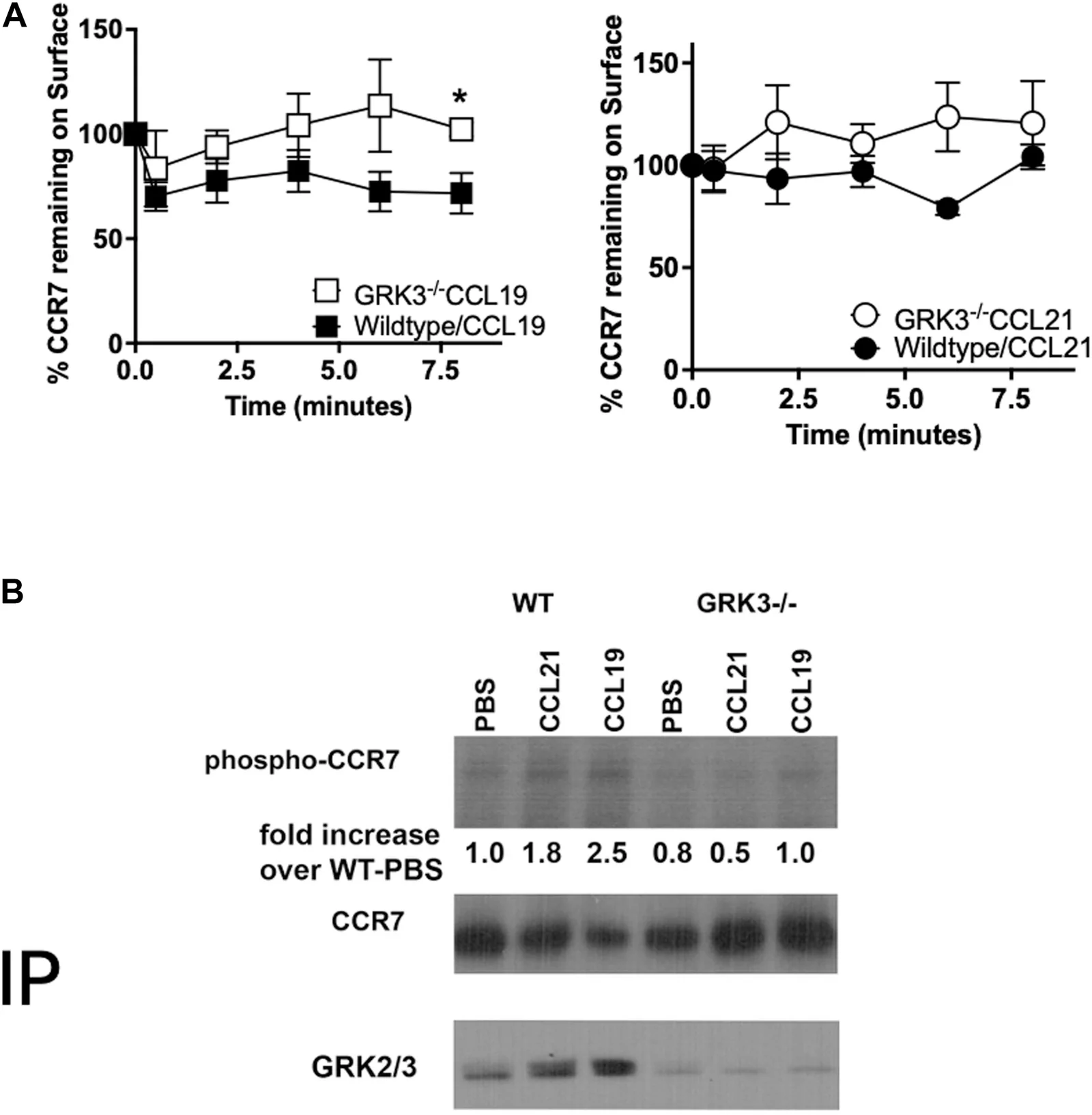
**GRK3 promotes phosphorylation in murine T cells in response to CCL19 and CCL21.** Murine T cells were isolated from splenocytes of wild type and GRK3^−/−^ mice and were analyzed for CCR7 internalization and phosphorylation. *A*, T cells were stimulated with 200 nM CCL19 or 200 nM CCL21 for the indicated times, and surface levels of CCR7 were quantified by flow cytometry. Data represent the mean ± SD of three independent experiments. ∗*p* = 0.0373. *B*, cells were metabolically labeled with ^32^P-orthophosphate, stimulated for 8 min with PBS, 200 nM CCL19 or 200 nM CCL21, lysed and CCR7 was immunoprecipitated. Lysates were fractionated and either immunoblotted for GRK2/3, stripped and reprobed for CCR7 or gels dried and analyzed by phospho-imaging. Levels of phosphorylated CCR7 were normalized to total immunoprecipitated CCR7 using ImageJ. Data are the means of two independent experiments. CCR7, C-C chemokine receptor 7.

Receptor phosphorylation occurs following ligand binding, contributing to receptor internalization. To define the role of GRK3 in regulating CCR7-mediated internalization, we compared ligand-dependent CCR7 internalization in splenic T cells from WT and GRK3^−/−^ mice in the presence of CCL19 or CCL21. Over an 8-min interval, GRK3^−/−^ cells failed to internalize CCR7 bound to CCL19, in contrast to WT cells, which internalized approximately 30% of surface CCR7 (Fig. 1*A*). In contrast, CCL21 induced limited internalization (∼7%) of CCR7 in WT cells. Notably, in GRK3^−/−^ T cells, the surface levels of CCR7 significantly exceeded the baseline levels (Fig. 1*A*) following stimulation with CCL19 (*p* = 0.0223) and CCL21 (*p* = 0.0383), which is consistent with the mobilization of CCR7 from intracellular stores (37, 38).

To determine if GRK3 promotes CCR7 phosphorylation, we measured ligand-mediated CCR7 phosphorylation in murine T cells. WT and GRK3^−/−^ T cells were metabolically labeled with ^32^P-orthophosphate-labeled murine and stimulated with CCL19, CCL21, or vehicle (PBS) for 8 min. CCR7 was immunoprecipitated and analyzed for phosphorylation levels after normalizing to the levels of immunoprecipitated CCR7. In WT cells CCL19 robustly increased phosphorylation of CCR7 when compared to CCL21 or vehicle (Fig. 1*B*). Notably, in primary mouse T cells, loss of GRK3 reduced CCL19-induced CCR7 phosphorylation in two independent experiments (Fig. 1*B*), suggesting that GRK3 contributes to CCL19-induced CCR7 phosphorylation. These observations in mouse T cells prompted us to determine whether GRK3 similarly regulates CCR7 phosphorylation in human T cells.

### GRK3 differentially regulates CCR7 phosphorylation but does not affect internalization in human T cells

**Figure 2:**
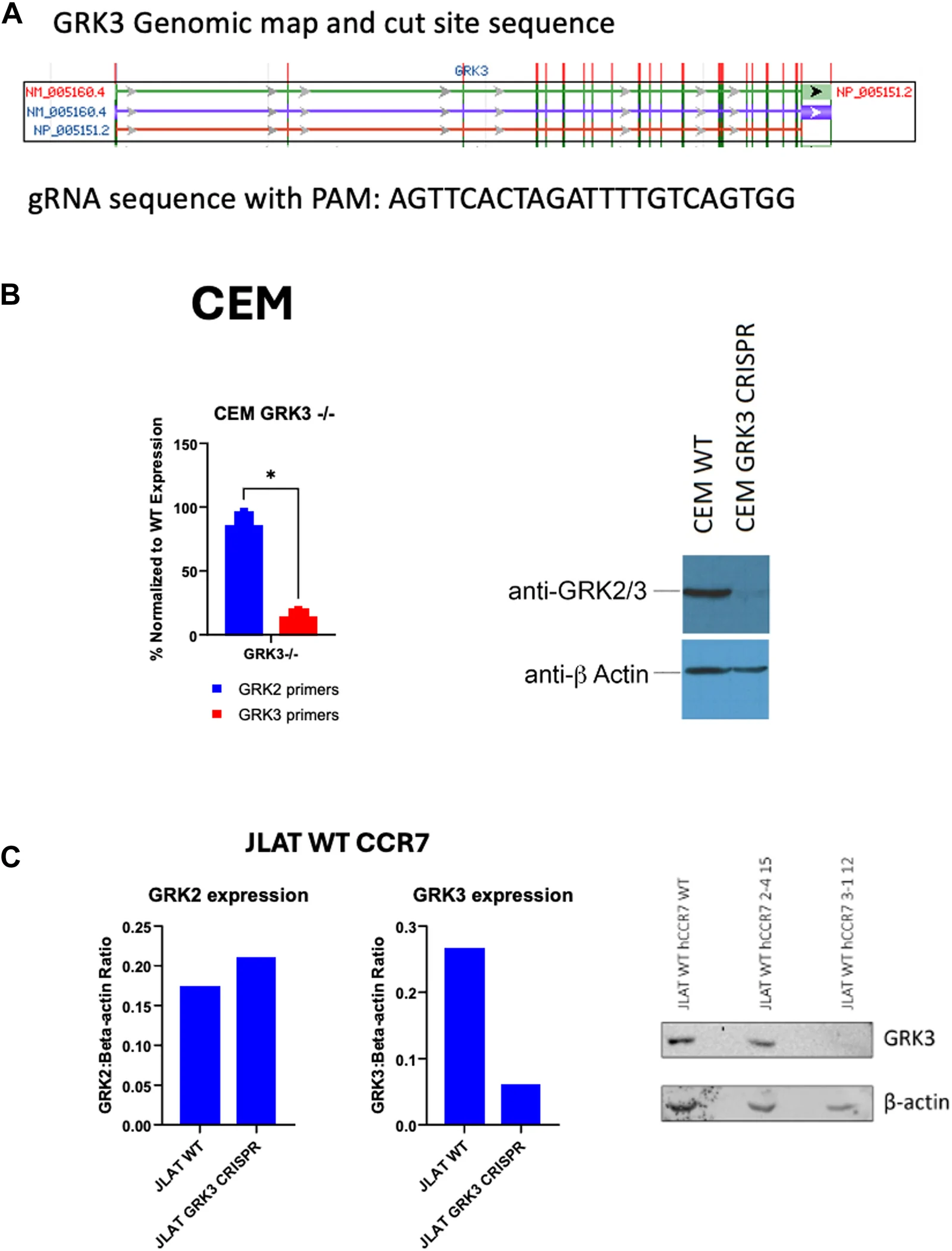
**CRISPR-Cas9 disruption of the GRK3 locus in human T cells.***A*, the full-length GRK3 gene (GenBank accession no. NM_005160.4) was scanned for PAM sequences and a guide RNA targeting GRK3 without overlap with GRK2 was designed. *B*, RT-PCR and western blot of whole cell lysates from wild-type CEM and GRK3^−/−^ CEM cells probed with anti-GRK2/3. *C*, RT-PCR and western blot of whole cell lysates from JLAT WT CCR7 and JLAT WT CCR7 GRK3^−/−^ cells probed with anti-GRK2/3. RNA was reverse transcribed and amplified, or cells were immunoblotted for GRK3, stripped and reprobed for actin as a loading control. CCR7, C-C chemokine receptor 7; PAM, protospacer adjacent motif.

To assess whether GRK3 similarly regulated CCR7 phosphorylation and internalization in human T cells, we used CRISPR-Cas9 to disrupt GRK3 expression in two human T cell lines. We used CEM cells, which endogenously express CCR7, and JLATWT-CCR7 generated by retroviral-mediated transduction of human CCR7-FLAG into the JLATWT Jurkat cells (36). Disruption of the GRK3 locus was verified by RT-PCR and western blot analysis of the GRK3-disrupted cell lines (Fig. 2, *A–C*). Notably, our RT-PCR results show that disruption of the GRK3 locus did not disrupt GRK2 expression.

**Figure 3:**
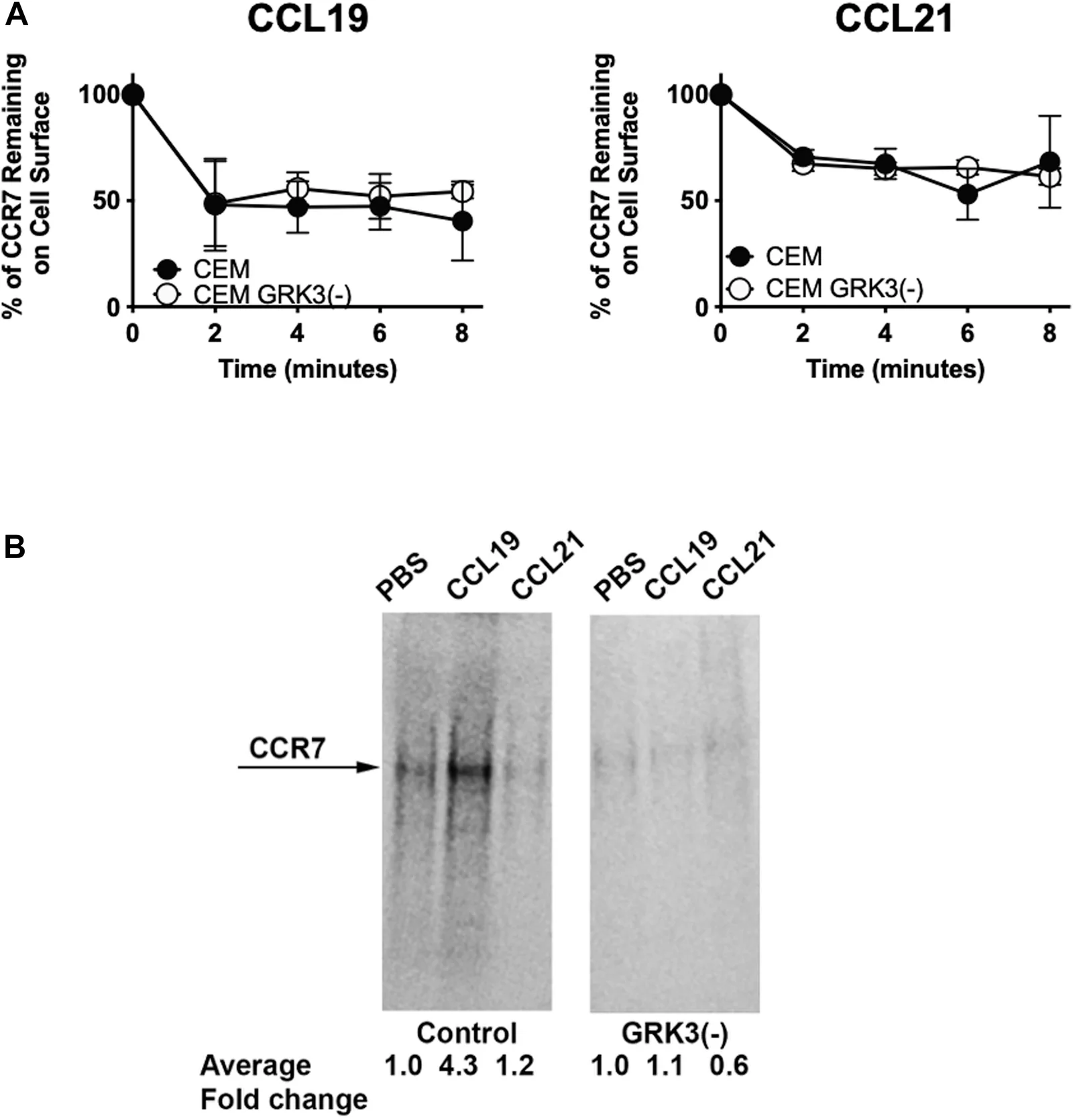
**GRK3 promotes CCR7 phosphorylation in human T cells in the presence of CCL19 and CCL21.** Human CEM T cells were analyzed for CCR7 internalization and phosphorylation. *A*, CEM cells were stimulated with 200 nM CCL19 or 200 nM CCL21 for the indicated times, and surface levels of CCR7 were measured using flow cytometry. Data represent the mean ± SD of three independent experiments. *B*, CEM cells were metabolically labeled in the presence of ^32^P-orthophosphate, stimulated with PBS, 200 nM CCL19 or 200 nM CCL21, lysed and CCR7 was immunoprecipitated. Lysates were fractionated, gels dried, and analyzed by phosphorimaging. Levels of phosphorylated CCR7 were normalized to levels found in unstimulated cells using ImageJ. Data are from the average of three independent experiments. CCR7, C-C chemokine receptor 7.

We consider that it is important to study the effects not only in clonal, immortalized cell lines, but also in primary T cells. In these studies, we used both WT and GRK3^−/−/^ primary murine T cells. In contrast to primary murine T cells, loss of GRK3 expression had a modest effect on CCR7 internalization in human T cells. CCL19 induced internalization of approximately 60% of CCR7 in WT cells and ∼ 48% in the GRK3 deficient T cells. Unlike primary murine T cells, CCL21 promoted internalization in human T cells, and this was unaffected by GRK3 disruption (Fig. 3*A*).

We then examined whether GRK3 regulates phosphorylation of CCR7 in human T cells. In CEM CCL19 promoted significantly higher levels of CCR7 phosphorylation than in CEMGRK3(−) T cells (*p* = 0.0361). Conversely, CCL21 stimulation of CEMGRK3(−) T cells phosphorylation below baseline, mirroring observations in murine T cells (Fig. 3*B*). These data from these studies indicated that in human T cells, GRK3 selectively promoted phosphorylation of human CCR7 downstream of CCL19 and CCL21.

### *GRK3* phosphorylation of CCR7 selectively alters CCL19-induced *Gαi3/*Gγ2 FRET

Activation of chemokine receptors promotes GDP to GTP exchange on Gαi within heterotrimeric G-proteins, triggering activation-associated rearrangement of the Gαiβγ complex. To determine if GRK3-mediated CCR7 phosphorylation affected assembly of Gαi/Gβγ with CCR7 we used FRET assays in JLAT WT-CCR7 and JLAT WT-CCR7GRK3(−). The Gαi (Gαi_1,_ Gαi_2_ or Gαi_3_) subunit was fused to mTurquoise2, which was co-expressed with cpVenus-Gγ2. Changes in FRET (ΔFRET) was calculated as the average change from baseline during the 8-min interval following ligand stimulation.

**Figure 4:**
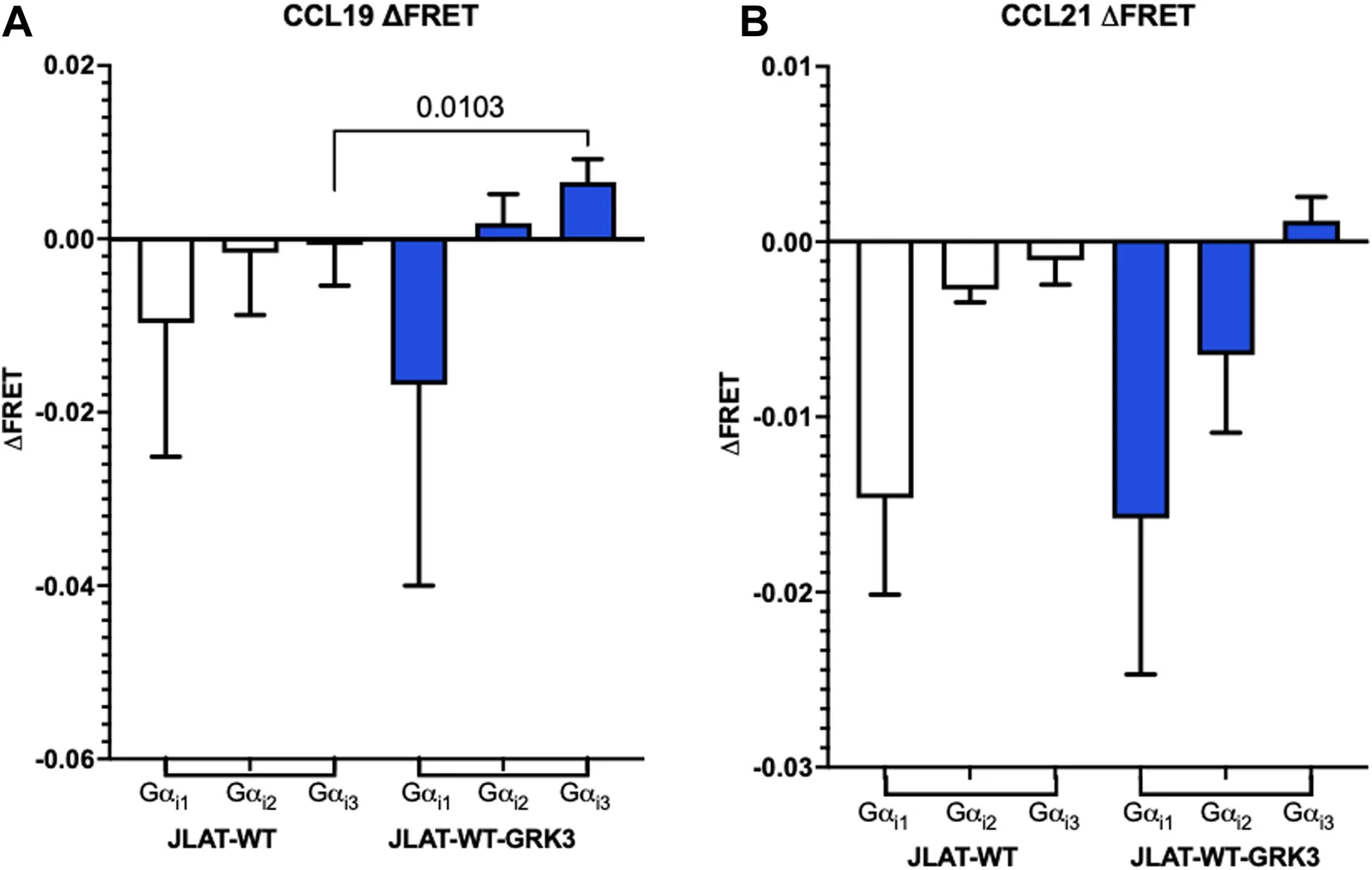
**GRK3 limits CCR7 recruitment of Gαi3 following binding of CCL19.** JLATWT-CCR7 or JLATWT-CCR7-GRK3(−) cells were nucleofected with Gαi (Gα_i1-_ mTurquoise, Gα_i2-_mTurquoise, or Gα_i3-_ mTurquoise) and cpVenus-Gγ2. FRET measurements were collected for 5 min prior to ligand addition to establish a baseline, and for 10 min following stimulation with CCL19 or CCL21. ΔFRET was calculated as the change in mean fluorescence in stimulated cells relative to baseline. Histograms are representative of four independent experiments run in duplicates ± SD. CCR7, C-C chemokine receptor 7.

In the absence of GRK3, CCL19 induced a significant increase in ΔFRET between Gαi_3_ and Gγ2, whereas CCL19 had no significant effect on ΔFRET between Gαi1 or Gαi2 and Gγ2. . Conversely, CCL21 did not differentially affect ΔFRET for any Gαi isoform in the presence or absence of GRK3 expression (Fig. 4). These findings indicate that GRK3 selectively regulates the CCL19-induced Gαi_3_/Gγ2 FRET downstream of CCR7 activation. Because changes in Gαi/Gγ2 FRET report activation-associated rearrangement of the heterotrimeric G protein, these data suggest that GRK3 selectively modulates Gαi3/Gγ2 FRET in response to CCL19 activation of CCR7.

### GRK3 limits CCR7-induced chemotaxis and Ca^2+^ mobilization

**Figure 5:**
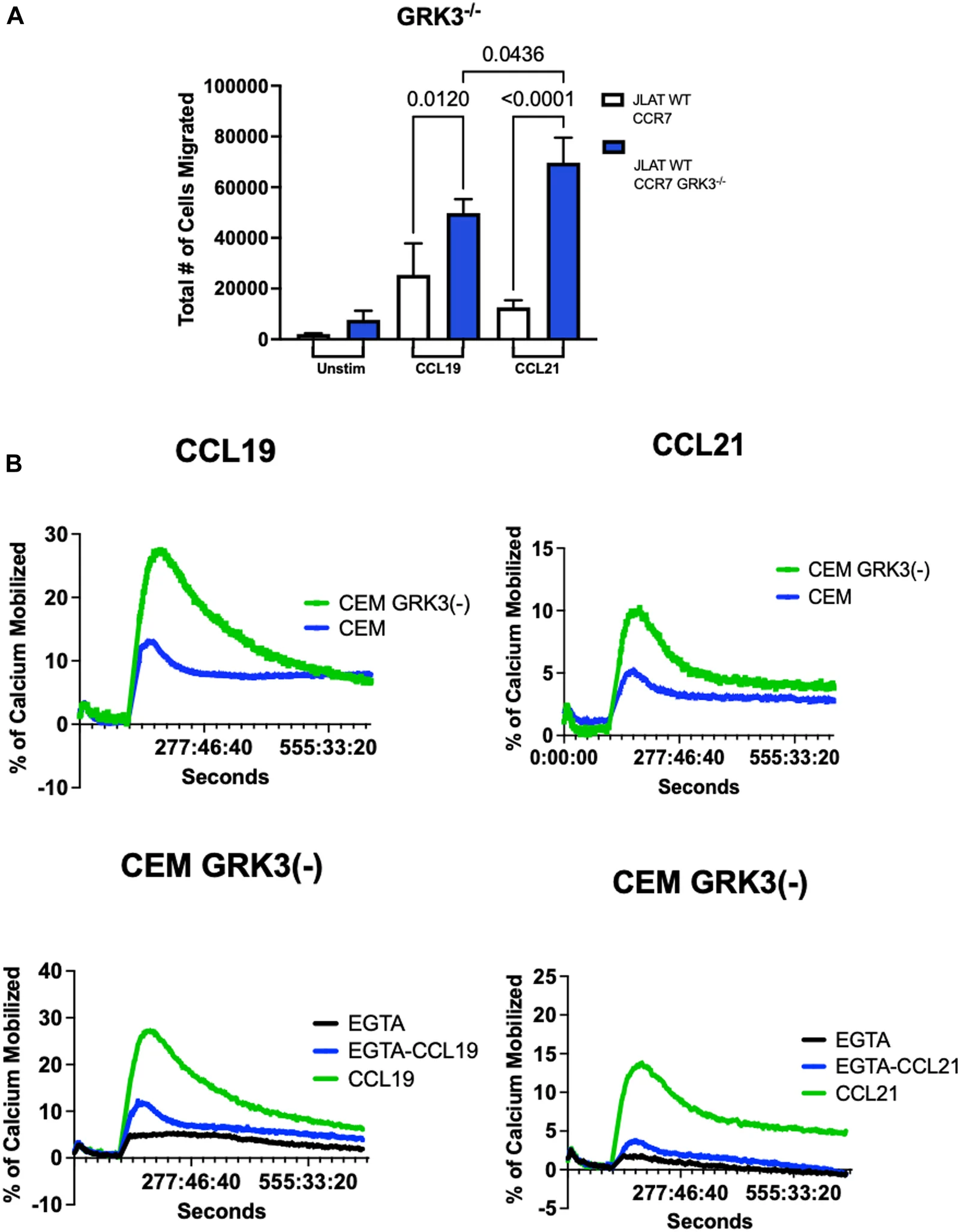
**GRK3 limits CCR7-mediated chemotaxis and Ca^2+^ mobilization.** JLATWT-CCR7 or JLATWT-CCR7-GRK3(−) cells were stimulated with 200 nM CCL19 or CCL21. *A*, cells were induced to migrate through fibronectin-coated transwell membranes for 90 min and migrated cells were quantified. Data are representative of three independent experiments run in duplicate ± SD. *p* values were determined from ANOVA analysis. *B*, cells were preloaded with Fura-4 and stimulated with CCL19 or CCL21. Intracellular Ca^2+^mobilization was measured over 10 min and normalized to ionomycin-mediated Ca^2+^mobilization at the termination of the assay. Data for each condition are an average of three independent experiments. CCR7, C-C chemokine receptor 7.

Because Gαi subunits mediate chemotaxis *via* chemokine receptors (39, 40), we questioned whether enhanced recruitment of Gαi_3_ in GRK3^−/−^ human T cells, would alter T cell chemotaxis (Fig. 5). Using fibronectin-coated membranes, we assessed chemotaxis of JLATWT-CCR7 and JLATWT-CCR7GRK3(−) cells over 90 min. GRK3 significantly limited transwell migration to CCL21, while inhibition of migration toward CCL19 was less pronounced (Fig. 5*A*).

Since immune cell chemotaxis is linked to Ca^2+^/calmodulin-mediated activation of myosin light chain kinase (41) we investigated whether GRK3 regulated Ca^2+^mobilization downstream of ligand binding. Both CCL19 and CCL21 promoted rapid Ca^2+^ responses in parental CEM cells following CCR7 binding to ligand; however, these responses were markedly increased in the GRK3^−/−^ cells. Indeed, peak Ca^2+^ mobilization in GRK3^−/−^ cells was greater that in parental CEM cells that express GRK3, with a strong trend toward significance (*p* = 0.0547). Chelation of extracellular Ca^2+^ with EGTA substantially reduced Ca^2+^ mobilization in CEM cells indicating reliance primarily on extracellular Ca^2+^ influx, with a smaller contribution from intracellular stores. Collectively, these findings demonstrate that GRK3 limits CCR7-dependent Ca^2+^ signaling and chemotactic responses.

### GRK3 slows arrestin-3 recruitment to CCR7 downstream of CCL19

Because GRK3 differentially regulated CCR7 phosphorylation and Gα_i3_ signaling, we tested whether GRK3 also affected arrestin-3 recruitment. We have previously shown that arrestin-3, but not arrestin-2 mediate CCR7 internalization downstream of CCL19 but not CCL21 (26). To visualize arrestin recruitment we coexpressed CCR7-GFP with arrestin-3 fused at the C-terminus to a hemagglutinin epitope tag (YPYDVPDYA) (Arr3-HA) in HEK293T cells.

**Figure 6:**
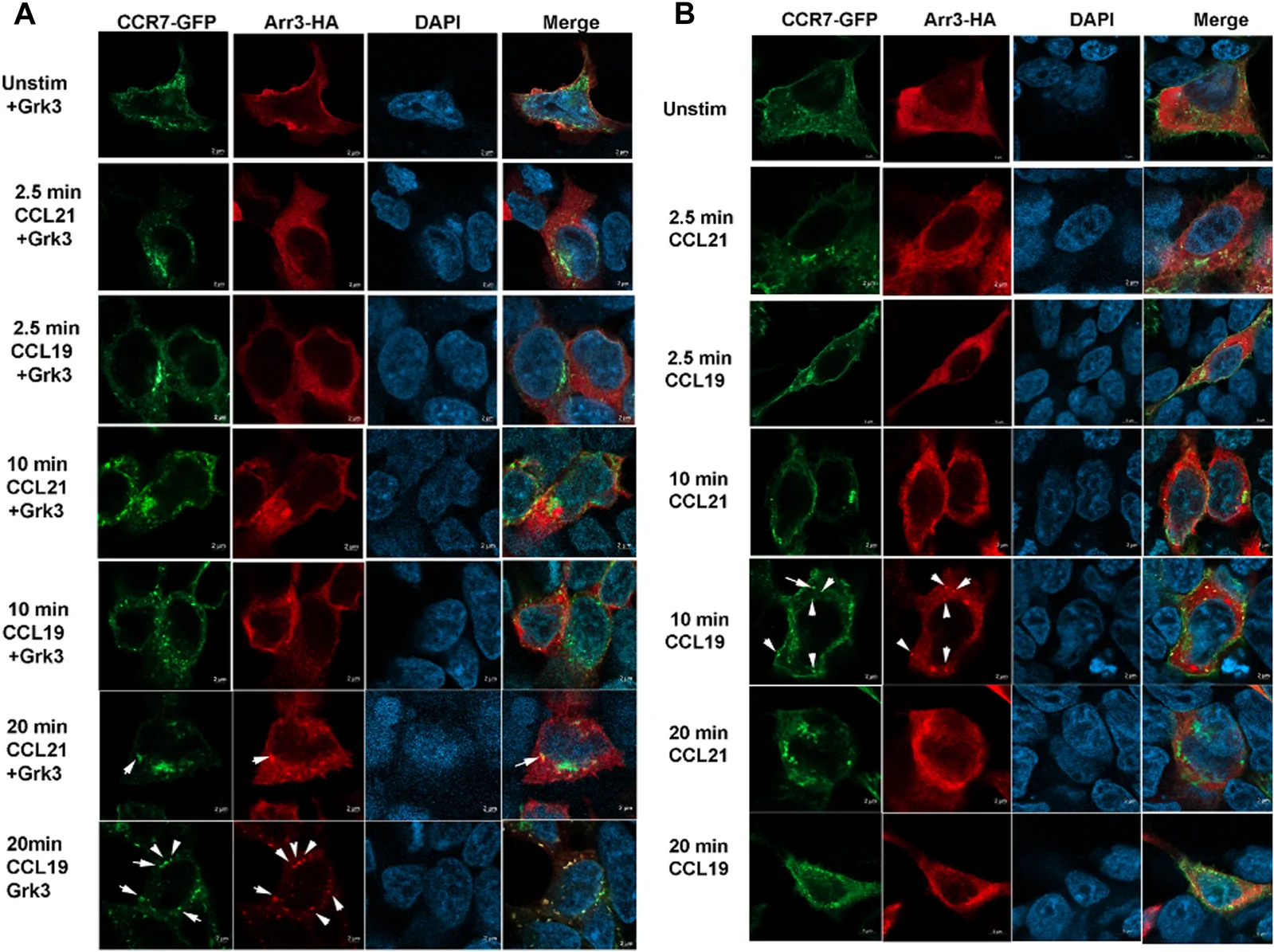
**GRK3 delays arrestin-3 recruitment to CCR7 following stimulation with CCL19.** HEK293T cells were transfected with CCR7-GFP, HA-tagged arrestin-3 (Arr3-HA) A) with control plasmid or B) with GRK3. Twenty-four hours later cells were stimulated with CCL19 or CCL21 the indicated times, fixed, and stained for HA and DNA (DAPI). Coverslips were mounted and imaged using a Zeiss LSM 900 confocal with Airyscan. Images are representative of a minimum of 50 cells per condition. CCR7, C-C chemokine receptor 7; DAPI, 4′,6-diamidino-2-phenylindole.

Consistent with previous findings (26), CCL19 induced detectable colocalization of CCR7 with arrestin-3 within 10 min, which was more apparent after 20 min of stimulation, whereas CCL21 failed to induce detectable CCR7-arrestin-3 colocalization (Fig. 6). In cells overexpressing GRK3, CCR7-arrestin-3 colocalization was not readily apparent at 10 min but was observed after 20 min of CCL19 stimulation (Fig. 6). These observations suggest that GRK3 limits time it takes for arrestin-3 recruitment of CC19-stimulated CCR7.

## Discussion

The goal of this study was to define the contribution of GRK3 to biased CCR7 signaling initiated by its two ligands, CCL19 and CCL21, and to determine how GRK3-dependent phosphorylation influences downstream signaling events that ultimately regulate chemotactic migration of T cells. CCR7 is well known to exhibit ligand bias, including differential recruitment of arrestin-3, which has been implicated in shaping signaling outputs and migratory behavior (26). Although GRK3 has previously been shown to phosphorylate CCR7 in response to CCL21, its role in regulating CCR7 phosphorylation in T cells in response to CCL19 has remained unclear. Our findings reveal an unexpected species-specific difference: GRK3 contributes to CCR7 phosphorylation downstream of CCL19 in murine T cells and in human T cells, highlighting an important convergence between murine and human CCR7 regulation that should be considered when extrapolating mechanistic models across species. Specifically, human and mouse CCR7 share 86% amino acid identity, and thus are well conserved. Although there is a strong overlap between human and murine CCR7 signaling events in T cells, some differences are striking such as the extent of ligand-receptor internalization (26, 27).

Phosphorylation of G protein–coupled receptors is classically associated with desensitization through steric inhibition of G-protein coupling, a process most often attributed to binding of arrestin-2 or arrestin-3 (β arrestins) (42, 43). However, our data suggest that GRK3-mediated phosphorylation of CCR7 may regulate receptor signaling through mechanisms that extend beyond canonical arrestin-dependent desensitization. Although we did not detect recruitment of arrestin-3 to CCR7 following CCL21 stimulation and have previously shown that CCL21-driven chemotaxis of T cells is independent of β arrestins (26), GRK3 nonetheless exerted a substantial influence on Gαi engagement and chemotaxis responses. These observations raise the possibility that GRK3 phosphorylation alters the conformation or accessibility of CCR7 intracellular domains in a manner that selectively shapes G-protein coupling, rather than globally terminating signaling.

Consistent with this model, loss of GRK3 resulted in ligand-dependent changes in the association of Gαi subunits with CCR7. In the absence of GRK3, Gα_i3_ association with CCR7 modestly increased following CCL21 stimulation and increased more robustly following CCL19 stimulation, yet these changes did not correspond to enhanced Gαi activation as measured by a decrease in ΔFRET. In contrast, GRK3 promoted activation of Gα_i2_ downstream of CCL21, as reflected by a decrease in ΔFRET in JLATWT-CCR7 cells. This decrease is consistent with increased dissociation of Gα_i2_ from the membrane during receptor activation and correlated with enhanced chemotactic responses. One speculative interpretation is that GRK3-dependent phosphorylation stabilizes a CCR7 conformation that favors productive coupling to Gα_i2_ while simultaneously restricting engagement of Gα_i3_, thereby biasing the qualitative output of CCR7 signaling in a ligand-dependent manner.

Because T cells predominantly express Gα_i2_ and Gα_i3_ (34, 35), these findings suggest that GRK3 may function as a molecular switch that fine-tunes CCR7 signaling by differentially regulating Gαi subtypes responses. GRK3-mediated phosphorylation of CCR7 could alter the receptor conformation or its interactions with heterotrimeric G proteins, thereby influencing the ability of individual Gα_i_ subtypes to undergo ligand-induced activation. In the absence of GRK3, the altered receptor phosphorylation state may preferentially affect the Gα_i3_ response to CCL19.

This possibility is supported by our FRET analyses, which revealed minimal changes in Gα_i2_/Gγ2 FRET signal but a much larger increase in Gα_i3_/Gγ2 FRET following CCL19 stimulation in the absence of GRK3. Because this assay reports changes in the relative configuration of Gα_i_ and Gγ2 associated with G-protein activation, rather than direct Gai association with CCR7, the increased Gα_i3_/Gγ2 FRET does not by itself establish enhanced recruitment of Gα_i3_ to CCR7. Instead, these findings suggest that GRK3 selectively regulates the CCL19-induced Gα_i3_ response downstream of CCR7 activation. Thus, one possibility is that GRK3-mediated phosphorylation alters the relative accessibility or conformational compatibility of CCR7 with different Gαi subtypes. Importantly, these findings suggest that GRK3 selectively regulates the subtype-specific Gα_I_ response to CCL19-bound CCR7.

The effects of GRK3 on CCR7 signaling appear to be immune cell–type specific. Previous studies in GRK3-deficient mice showed no difference in chemotactic migration of neutrophils toward either CCR7 ligand, whereas dendritic cells exhibited enhanced migration toward CCL19 in the absence of GRK3 (32, 44). In those studies, GRK3 was proposed to limit dendritic cell migration by promoting receptor desensitization. Notably, the role of GRK3 in regulating responses to CCL21 was not examined, as CCL21 forms immobilized gradients *in vivo* through interactions with matrix proteins. In contrast, our examination of T cell migration toward static concentrations of both CCL19 and CCL21 revealed that GRK3 limits chemotaxis toward both ligands. These findings suggest that CCR7-mediated migratory signaling in T cells shares features with dendritic cells that are distinct from neutrophils, highlighting the importance of cellular context in shaping GRK3 function.

Beyond signaling at the plasma membrane, receptor internalization represents an additional layer of CCR7 regulation. We found that CCR7 was rapidly internalized in response to CCL19 regardless of GRK3 expression, suggesting that GRK3-mediated phosphorylation is not required for arrestin-dependent internalization in T cells. This prompted us to examine whether GRK3 influences post endocytic trafficking of CCR7. Using HEK293T cells, we found that overexpression of GRK3 delayed trafficking of CCL19-internalized CCR7 to arrestin3-positive endosomes, while having no effect on trafficking of CCL21-internalized receptors. These results suggest that although GRK3 phosphorylation is dispensable for CCR7 internalization, it may facilitate receptor trafficking within the endosomal compartment. In the absence of GRK3, CCR7 may recycle more rapidly to the cell surface following CCL19 stimulation, thereby limiting effective receptor downregulation. In contrast, because CCL21-induced CCR7 internalization does not rely on arrestin3, GRK3-mediated phosphorylation may promote arrestin-independent internalization. This process appears to be particularly important for limiting T cell migration toward static CCL21 gradients, as GRK3-deficient cells exhibited significantly enhanced migration.

Together, our findings identify GRK3 as a critical regulator of biased CCR7 signaling in T cells, acting at multiple levels to shape G protein coupling, receptor trafficking, and chemotactic responses. Although certain signaling outputs, such as Ca^2+^ mobilization, were unaffected by GRK3 loss, others were selectively regulated, underscoring the specificity of GRK3 action. Dysregulated CCR7 signaling has been linked to autoimmune diseases including Crohn’s disease and Hashimoto’s thyroiditis (45, 46, 47, 48, 49), and loss of CCR7 or its ligands results in exaggerated yet delayed secondary immune responses (50, 51, 52). A deeper understanding of how GRK3 regulates ligand-biased CCR7 signaling may therefore provide opportunities to selectively modulate pathological immune activation while preserving homeostatic immune surveillance.

## Experimental procedures

### Mice

The mice were a generous gift from Dr Robert Lefkowitz (Duke University, Durham, NC). The mice were bred in house and genotyped as described (53) at the University of Kansas Medical Center (KUMC). All procedures were carried out in compliance with the KUMC-IACUC approved protocol 2010-1876. Briefly, mice were euthanized, and spleens were collected. T cells were isolated from splenocytes using the EasySep Pan Naïve T Cell Isolation Kit (#19848) and used in the internalization and metabolic labeling studies.

### Antibody validation

ALEXA Fluor 700 anti-human CCR7 (#353243; Bio-Legend) and anti-hCCR7 (R&D Clone # 150503) were validated by using flow cytometry to compare expression of CCR7 levels on Jurkat which do not endogenously express CCR7 to CEM and HuT78, which express CCR7 endogenously. Anti-murine CCR7 (4B12) was validated by comparing CCR7 expression in primary murine WT T cells to CCR7^−/−^ T cells isolated from spleens of C57BL/6 mice anti-HA-ALEXA Fluor 647(2–2.2.14) was used on HEK cells in the presence or absence of HA-Arrestin 3. Anti-mouse GRK3, anti-beta actin, anti-FLAG and anti-S/T were validated by the suppliers.

### Cell lines

The CEM human T cell lymphoblastic leukemia cell line was a generous gift from Dr Iannis Aifantis (New York University) and were validated by American Type Culture Collection (ATCC), and JLAT-WT, were a generous gift from Dr Arthur Weiss (University of California at San Francisco, San Francisco). JLATWT-CCR7 were generated by transduction of pGEN-Lenti-CCR7 (GenScript), which encodes an 8-amino acid linker, fused at the C terminus to FLAG (DYKDDDDK). HEK293T cells were purchased from the ATCC and were validated by ATCC. CEM and JLATWT cells and derivative lines were cultured in heat-inactivated 10% fetal bovine serum (R&D Systems)/90% RPMI (Corning)/2 mM L-glutamine (Invitrogen). Prior to transduction with CCR7, JLATWT was supplemented with 1.4 mg/ml geneticin (G418) to maintain LAT expression in cells (54). HEK293T cells were maintained in heat-inactivated 10% fetal bovine serum (R&D)/90% (Dulbecco's modified Eagle's medium) DMEM/2 mM L-glutamine (Invitrogen). All cell lines were cultured in a humidified atmosphere of 5% CO_2_/air at 37 °C. All cell lines were tested for *mycoplasma* and were found to be *mycoplasma* free.

### Receptor internalization assay

One million cells were resuspended in serum free RPMI (*sf*RPMI) and allowed to equilibrate in a 37 °C water bath for 10 min. Subsequently, 2 × 10^5^ cells were removed prior to addition of ligand and used for the 0-min time point and unlabeled control. Ligand was adjusted to 200 nM and receptor allowed to internalize for the indicated times. At each time point 2 × 10^5^ cells were transferred to 1 ml of ice-cold PBS. Following CCR7 internalization, cells were incubated on ice for at least 10 min, pelleted by centrifugation (90*g*, 4 °C for 5 min). The cells remained on ice for the remainder of the staining. Supernatants were removed, cells were rinsed 2 times in *sf*RPMI and stained for 20 min with ALEXA Fluor 700 anti-human CCR7 (#353243; Bio-Legend), or isotype controls from the same manufacturer for 30 min on ice in Ca^2+^/Mg^2+^ free 1× PBS/2% bovine serum albumin (BSA) (#A3059–100G, Sigma-Aldrich). Cells were rinsed twice in Ca^2+^/Mg^2+^ free 1× PBS and assayed immediately by flow cytometry on a Gallios Flow Cytometer (Beckman Coulter). The level of receptor remaining on the cell membrane was calculated as [mean channel fluorescence of conjugate/anti-CCR7 at time point]/[mean channel fluorescence of conjugate/anti-CCR7 at time = 0 min]. Statistical significance was determined using a paired Students *t* test. Data were fit using the Prism 7 software package (Graphpad Prism).

### pLenti-CRISPR construction for GRK3

The Genomic-scale CRISPR Knock-Out (GeCKO) lentiviral CRISPR vector (#52961; Addgene) was purchased from Addgene and used to generate the CRISPRs (55, 56). Briefly, GRK3 genomic sequence exons were scanned for the NGG protospacer adjacent motif (PAM). To target specifically GRK3 sequences, we eliminated sequences that overlapped GRK2 and GRK3. Forward oligonucleotides flanked the 5′ end of the 20 NT target sequence immediately 5′ of the NGG PAM with CACCG(CACCGagttcactagattttgtcag). The complementary reverse oligonucleotides were initiated with AAAC fused to the complementary strand and terminated with dCTP (AAACctgacaaaatctagtgaactC). The oligos were annealed and phosphorylated with T4 PNK (New England Biolabs (NEB)) and ligated into pLenti-CRISPR v2 that had been predigested with BsmBI to generate pLenti-CRISPRv2-Grk3. Other guide RNAs were used, and we selected this target since it most efficiently knocked down Grk3 with minimal effects on GRK2 expression.

### Lentiviral production

HEK293T cells were grown to 70% confluency in 10% heat inactivated fetal bovine serum (R&D)/90% DMEM(Corning)/2 mM L-glutamine (Invitrogen) (cDMEM) and cotransfected using LipoD293 (SignaGen Laboratories), with pVsVg, pD8.9, and the coding vector, pLentiCRISPR-GRK3 (Addgeneor pGen-Lenti-hCCR7-FLAG (GenScript). Twenty-four hours later, cells were rinsed with PBS with Ca^2+^/Mg^2+^, fed with cDMEM and supernatants collected 48 h later. Lentiviral containing supernatants were concentrated using Lenti-X (Takara Bio). CEM cells were transduced with pLentiCRISPR-GRK3 by spinoculation at a multiplicity of infection of 100, 250, 500, 750, and 1000 (MilliporeSigma Spinoculation protocol). Transduced CEM or JLATWT cells were incubated for 72 h and selected for transduction in 250 ng/ml (CEM) or 400 ng/ml (JLATWT) Puromycin/10% fetal bovine serum/RPMI. JLATWT-hCCR7 were incubated for 72 h and selected for CRISPR transduction in 2 ng/ml Puromycin/200 μg/ml Zeocin/10% fetal bovine serum/RPMI, expanded and propagated. Several CRISPRs were synthesized. DNA was isolated from pLentiGuide-GRK3-Puro CEM, the targeted locus amplified by PCR and sequenced. CCR7 expression was confirmed by flow cytometry.

### Western blot

Cells were washed three times in PBS (Ca^2+^/Mg^2+^ free) and stimulated with 200 nM CCL19 or 200 nM CCL21 in serum-free RPMI (*sf*RPMI) for the indicated times. The cells were lysed in 1× RIPA buffer (25 mM Tris–HCl pH 7.6, 150 mM NaCl, 1% NP-40, 1% sodium deoxycholate and 0.1% SDS, 50 mM NaF, 100 μM NaVO_4_, 40 μM sodium pyrophosphate, and 25 mM β-glycerophosphate) and supernatants cleared at 14,000*g* at 4 °C, mixed with 4× Laemmli, and fractionated by SDS-PAGE. Lysates were transferred to polyvinylidene fluoride (PVDF), membranes were blocked with 3% milk or BSA in TBS-T for 30 min and incubated with primary antibodies overnight at 4 °C using the following dilutions: mouse anti-GRK2/3 (1:1000; Merck Millipore), mouse anti-GRK3 (1:1000, Cell Signaling Technology), anti-beta actin HRP (1:1000, Cell Signaling), rabbit anti-DYKDDDDK (1:1000, Cell Signaling) and rabbit pS/T (1:1000, Invitrogen). For unconjugated antibodies, membranes were washed three times in TBS-T and incubated with anti-mouse or anti-rabbit HRP at (1:10,000; Jackson Laboratory) in 3% milk or BSA in TBS-T for 1 h at room temperature. Membranes were rinsed three times in TBS-T and developed with Super Signal West Pico PLUS and captured with iBright Imaging Systems (Thermo Fisher Scientific) or by exposure to X-ray film and developed.

### Immunoprecipitation of CCR7

Briefly, T cells (EasySep Pan-T isolation kit) were isolated from either WT or GRK3^−/−^ splenocytes. Cells were counted and 10^7^ T cells were grown in the presence of 500 μCi [^32^P] orthophosphate (Revvity Health Sciences)/10% dialyzed serum (against phosphate free RPMI, to remove phosphates)/90% phosphate free RPMI/2 mM L-glutamine overnight in a humidified environment @37 °C/5%CO_2_/air. For the final 15 min of the incubation, media concentrations were adjusted to 100 nM LF microcystin. Cells were collected by centrifugation, resuspended in phosphate free RPMI and stimulated with 200 nM of murine CCL19, CCL21 in PBS, or PBS alone for 10 min or treated with an equal volume of PBS (control) @37 °C. Cells were washed twice with PBS, and lysed in 500 μl of Brij buffer (10 mM Tris–HCl (pH 7.5), 150 mM NaCl, 1 mM EDTA, 1% Brij 96, 4 mM p-nitrophenyl phosphate, 40 mM sodium phosphate, 40 mM NaF, Sigma protease inhibitor cocktail, 50 mM β-glycerophosphate, 100 μM Sodium orthovanadate, 100 nM microcystin-LF) for 20 min on ice. Lysates were clarified by centrifugation, and CCR7 immunoprecipitated with 2 μg anti-murine CCR7 Thermo Fisher Scientific (clone 4B12)/30 μl protein Pierce A/G beads (Thermo Fisher Scientific) for mouse cells, or 2 μg anti-hCCR7 (R&D Clone # 150503) at room temperature for 1 hour followed by a 16-h incubation at 4 °C, with rotation; immune complexes were isolated by centrifugation for 20 s and rinsed two times with fresh 1% Brij lysis buffer. The beads were separated from the lysate by the addition of 40 μl 2× Laemmli sample buffer. Equal volumes of immune complexes were fractionated on 4 to 15% gradient acrylamide gels (Bio-Rad Laboratories), by SDS/PAGE, gels were dried exposed to X-ray film and relative amounts of ^32^P were determined using ImageJ software or transferred to polyvinylidene fluoride overnight, stained and probed for CCR7.

### Nucleofection

For the fluorescence resonance energy transfer (FRET) studies (48), 2 × 10^6^ JLATWT-CCR7 cells were nucleofected with plasmids purchased from Addgene (generous gifts from Dorus Gadella). In addition, 0.25 μg of Qiagen purified Gαi subunit ((Gαi_1_-mTurquoise2 (#69620), Gαi_2_-mTurquoise2 (#69621), or Gαi_3_-mTurquoise2 (#69622)) was cotransfected with 0.25 μg G-protein Gγ2, tagged with circularly permutated Venus (cpVenus-Gγ2 (#69626) in Optimem (Gibco #31985062). Following nucleofection, cells were allowed to rest for 10 min and then transferred to a 6-well dish and complete RPMI media.

### Fluorescence resonance energy transfer

Transfected JLATWT-CCR7 and JLATWT-CCR7 GRK3^−/−^ cells were resuspended at 2.0 × 10^6^ cells/ml in Hanks balanced salt solution (HBSS), plated on black 96-well plates at 100 μl (2.0 × 10^5^ cells) per well and measured using a Fluoroskan Ascent FL (Thermo Fisher Scientific) following stimulation with 50 nM of CCL19 or CCL21 or PBS control. Donor emission was measured at 490 nM and acceptor emission at 550 nM every second per well, allowing cells to generate a baseline measurement for 2 min, and stimulation for 6 min. Differences between donor and acceptor emission were plotted as mean fluorescence intensity (MFI) for each Gαi subunit. ΔFRET was calculated as the difference between the Mean MFI _FRET_-Mean MFI _Baseline_.

### Transwell chemotaxis assays

JLATWT-CCR7 and JLATWT-CCR7 GRK3^−/−^ cells were washed, resuspended in HBSS buffer with 1% BSA, and resuspended at a concentration of 4 × 10^6^/ml. The lower chambers of Neuro Probe AP48 transwell chambers were filled with 30 μl CCL19, CCL21, or no ligand at 50 nM. Fibronectin-coated (Sigma-Aldrich, FC010) 5 μm polycarbonate membranes (10 μg/ml) were placed over the chemokines in a Neuroprobe chemotaxis chamber, and the lid secured. Fifty microliters of the cell suspension (2 × 10^5^) was loaded into the top well and cells allowed to migrate for 3 h at 37 °C in a 5% CO2 humidified incubator. Cells in the lower well were counted and results normalized to the number of cells that migrated with ligand alone.

#### Calcium mobilization assay

Cells were washed in HBSS and resuspended at a 5 × 10^6^/ml concentration. Fluo-4 AM at a concentration of 4 μM and Pluronic F-127 at 4.4% were added to cells to stain them for 45 min at 37 °C. After incubation, cells were washed with HBSS and then incubated at room temperature for 45 min in the dark. Once stained, the cells were resuspended in HBSS/2 μM EGTA or HBSS alone, at a concentration of 4 × 10^5^/100 μl. Conditions tested were normalized to ionomycin 2 μM with duplicates for each condition. Cells were plated on black 96-well plates and measured on Fluoroskan Ascent FL (Thermo Fisher Scientific) by stimulating cells with 200 nM of CCL19 and CCL21 after baseline measurement. Fluo-4 AM excitation is at 485 nM, emission at 527 nM, baseline measurement for 2 min, and stimulation for 10 min. Experiments were performed in triplicate.

#### hCCR7-GFP

PCR amplified full-length human CCR7 from complementary DNA (cDNA) and fused to GFP in pEGFP-N1 (Qiagen). cDNA template for hCCR7 was reverse transcribed from mRNA template of CEM cells. The insert and backbone were ligated with T4 DNA ligase (NEB).

### Expression of GRK3 in the HEK cell line

HEK293T cells were grown to 70% confluency and cotransfected with plasmids *via* LipoD293 (SignaGen) manufacturer protocol. Briefly, plasmids HA-Arr3 (β-Arrestin 2) (Addgene #14692), hCCR7-GFP, and pGRK3 (a generous gift from Dr Marc Caron) were resuspended in DMEM, and LipoD293 diluted in a separate tube. DNA and lipid were combined, allowed to incubate for 10 min at room temperature, and transferred to individual wells of a 6-well dish. The next day, cells were plated onto 12 mm round coverslips coated with poly-L-Lysine at a concentration of 5.0 × 10^4^ cells per coverslip.

#### Fluorescence microscopy

Transfected HEK293T cells on coverslips were washed three times with PBS and then stimulated with 200 nM of CCL19 or CCL21 in serum-free DMEM at 37 °C for 0 min (unstimulated), 2.5 min, 10 min, or 20 min. Following incubation, cells were washed with PBS and fixed with 4% formaldehyde/PBS for 20 min. Fixed cells were permeabilized and blocked with 3% BSA in PBS/1% NP40 for 30 min. Cells were stained with 1:400 anti-HA-ALEXA Fluor 647(2–2.2.14) in the presence of 3% BSA in PBS for 60 min and washed 3 times with PBS @ 5 min/wash. Nuclei were stained with 4′,6-diamidino-2-phenylindole (DAPI) for 5 min at a dilution of 1:47,000 in PBS and excess removed with PBS. The coverslips were then mounted to microscope slides with ProLong Gold Antifade and cured for at least 12 h before imaging on a Zeiss 880 Airyscan.

## Data availability

The raw data provided in the manuscript are available upon request from Dr Vines (cvines@utep.edu) Phone 915-747-8127.

## Conflict of interest

The authors declare that they have no conflicts of interest with the contents of this article.
